# Optimism Is Prospectively Associated With Resilience During the COVID-19 Pandemic

**DOI:** 10.1093/geroni/igab046.1208

**Published:** 2021-12-17

**Authors:** William Chopik, Jeewon Oh, Mariah Purol, Eric Kim

**Affiliations:** 1 Michigan State University, East Lansing, Michigan, United States; 2 University of British Columbia, Vancouver, British Columbia, Canada

## Abstract

Emerging research has identified how protective factors—like optimism—are associated with resilience to stress during the COVID-19 pandemic. However, the majority of research is cross-sectional, which creates ambiguity around the causal direction because these very protective factors might have also changed due to the pandemic. In the current study, we used longitudinal data from the Health and Retirement Study (N = 921; Mage = 64.54, SD = 10.71; 59.6% female; 57.5% White) to examine how optimism measured in 2016 predicted adjustment during the pandemic (in 2020). Higher baseline levels of optimism were subsequently associated with less worrying and stress resulting from changes in social contacts (βs > |.10|), less loneliness and not feeling overwhelmed (βs > |.16|), and greater COVID-related resilience and benefit-finding (β = .21). The findings will be discussed in the context of mechanisms that facilitate the protective functions of optimism and other psychological characteristics.

